# The relationship between the interictal epileptiform discharge source connectivity and cortical structural couplings in temporal lobe epilepsy

**DOI:** 10.3389/fneur.2023.1029732

**Published:** 2023-02-01

**Authors:** Zhensheng Li, Che Jiang, Quwen Gao, Wei Xiang, Zijuan Qi, Kairun Peng, Jian Lin, Wei Wang, Bingmei Deng, Weimin Wang

**Affiliations:** ^1^Department of Neurology, General Hospital of Southern Theater Command, Guangzhou, China; ^2^Department of Neurosurgery, General Hospital of Southern Theater Command, Guangzhou, China; ^3^Department of Neurosurgery, Sun Yat-sen Memorial Hospital, Sun Yat-sen University, Guangzhou, China

**Keywords:** interictal epileptiform discharges (IEDs), source connectivity, cortical, temporal lobe epilepsy (TLE), structural couplings

## Abstract

**Objective:**

The objective of this study was to explore the relation between interictal epileptiform discharge (IED) source connectivity and cortical structural couplings (SCs) in temporal lobe epilepsy (TLE).

**Methods:**

High-resolution 3D-MRI and 32-sensor EEG data from 59 patients with TLE were collected. Principal component analysis was performed on the morphological data on MRI to obtain the cortical SCs. IEDs were labeled from EEG data and averaged. The standard low-resolution electromagnetic tomography analysis was performed to locate the source of the average IEDs. Phase-locked value was used to evaluate the IED source connectivity. Finally, correlation analysis was used to compare the IED source connectivity and the cortical SCs.

**Results:**

The features of the cortical morphology in left and right TLE were similar across four cortical SCs, which could be mainly described as the default mode network, limbic regions, connections bilateral medial temporal, and connections through the ipsilateral insula. The IED source connectivity at the regions of interest was negatively correlated with the corresponding cortical SCs.

**Significance:**

The cortical SCs were confirmed to be negatively related to IED source connectivity in patients with TLE as detected with MRI and EEG coregistered data. These findings suggest the important role of intervening IEDs in treating TLE.

## Introduction

Epilepsy is a chronic neurological disease characterized by episodes of abnormal neural activation that result in sensory, psychiatric, and neurological dysfunction. Temporal lobe epilepsy (TLE) is the most common form of epilepsy and is notorious for being pharmacoresistant (1). Recent findings indicate that neurobiological alterations in patients with a significant history of TLE are not limited to temporal areas but can extend to cortical regions (2). The cortical atrophy in patients with TLE has been mainly identified in the regions that connect with the temporal structures, including the prefrontal, limbic, and perisylvian regions. Some subcortical nuclei such as the thalamus and basal ganglia can also show atrophy in patients with TLE. Studies suggest that gray matter atrophy in TLE can be related to the structural connectivity of the temporal lobe (3). Alterations in structural connectivity also can cause a wide array of comorbidities in patients with TLE, including cognitive dysfunction, neuropsychological disorders, sleep disorders, and poor quality of life in patients with TLE (4, 5).

For diagnostic purposes, interictal epileptiform discharges (IEDs) are important pathological biomarkers detectable *via* electroencephalogram (EEG). In addition to being used to locate the epileptogenic focus for focal epilepsy, IEDs can also be applied for the analysis of brain activity dynamics (6) and obtaining information about functional brain connectivity (7). Nowadays, the findings of IED analysis reveal the epileptogenic network associated with clinical seizures and explain the presence of intrinsic brain network mechanisms of epilepsy (8, 9). Recent studies have also found that patients presenting with extensive structural and functional brain abnormalities are often closely associated with spreading IEDs (10). Therefore, uncovering and monitoring brain network alterations based on IED analysis are essential to discover structural hubs that can be used for the treatment that could control seizures effectively and protect patients' cognitive function. These networks can even be used as important structural biomarkers to predict the outcome of surgical treatment in patients and to discover new targets for micro-invasive therapies and neuromodulation therapy.

There have been many studies on patients with TLE about brain connectivity based on IEDs using novel computational algorithms; for example, the Epilepsy Connectome Project is gradually unfolding worldwide (11). However, there is a lack of integrated analysis between EEG data and cerebral structural imaging data for patients with TLE. The integrated analysis can potentially allow for precise detection of the origin and spreading pathways of epileptiform discharges, as well as an advanced understanding of the pathophysiology of TLE. Therefore, this study will perform a source localization analysis of IEDs in TLE and explore the relationship between the IED source connectivity with the cortical structural couplings (SCs). We aimed to test the hypothesis that the spreading patterns of IEDs in patients with TLE are associated with the corresponding cortical SCs on the pathophysiological level.

## Methods

### Participants

This study included 59 patients diagnosed with unilateral temporal lobe epilepsy (TLE) between 2015 and 2020. The inclusion criteria are as follows: patients with TLE, with a diagnosis confirmed *via* seizure semiology, interictal/ictal electroencephalogram, and video-telemetry recordings, as well as evidence of structural abnormalities in the temporal lobe on magnetic resonance imaging (MRI) according to the diagnostic criteria devised by the International League Against Epilepsy (ILAE) (12). Of these, 33 patients were diagnosed with unilateral hippocampal sclerosis and 15 of these underwent surgery, with the pathology indicating hippocampal sclerosis combined with focal cortical dysplasia and the seizure outcomes categorized as completely seizure-free (Engel class I), while 26 patients had negative MRI results or a slightly increased signal intensity in the hippocampal and amygdala structures. The study also included patients aged 16–40 years who were right-handed, while patients with TLE caused by inflammation, trauma, brain tumor, or vascular disease were excluded.

Written consent was obtained from 59 participants. One of the female patients was under 18 years of age, and consent was provided by both of her parents. This study was approved by the institutional review board at the General Hospital of Southern Theater Command.

### Neuroimaging procedures

#### Acquisition

All the participants diagnosed with TLE underwent a high-resolution brain MRI with a General Electric (GE) 3.0T MR scanner. The traditional T1-weighted, T2-weighted, T2 FLAIR axial sequences, the T2-weighted and T2 FLAIR coronal sequences along the hippocampal axis, and the 3D T1-weighted MPRAGE sequences were acquired with the following parameters: (TE = 3.24 ms, TR = 2,300 ms, TI = 900 ms, FA = 9 degrees, bandwidth = 210 Hz/pixel, FOV = 256 mm, matrix size = 256 × 256, resolution = 1.0 × 1.0 × 1.0 mm^3^, number of slices = 176, and sagittal acquisition).

#### Image processing

The 3D T1-weighted MPRAGE images were converted into NIFIT format and imported into the Linux workstation. The artifacts of image noise, inhomogeneity, and patient head movement were excluded to control image quality, and rescanning is required for the images with poor quality. FreeSurfer 5.3 software (https://surfer.nmr.mgh.harvard.edu) was used to segment, reconstruct, and parcellate the cortical surface and subcortical GM structures (hippocampus, amygdala, caudate nucleus, shell nucleus, pallidum, volar nucleus, and brainstem). The process involved removal of non-cerebral tissue; intensity normalization; Talairach transformation; segmentation of subcortical GM structures; tessellation of the gray matter (GM)–white matter (WM) interface and automated correction of topological defects; surface deformation toward the GM–WM interface following intensity gradients to construct the pial surface; and cortical parcellation was based on the Desikan–Killiany (DK) atlas (13).

### EEG procedures

#### Video-EEG acquisition

The video-EEG data were recorded with Bio-logic 32-sensor video-EEG monitoring equipment (Ceegraphy vision Digital EEG system, version 7.15.06. Copyright ©Bio-logic System Corp.). The sampling rate was set to 256–512 Hz, band-pass filtering was selected, the filter range was set to 0.5–70 Hz, the Notch was set to 50 Hz, and the time constant was set to 0.3 s. The 32 scalp sensors and bilateral sphenoid sensors were placed for the patients with TLE according to the international 10–20 system, and the average electrode was set as the reference electrode. The data were recorded continuously for 24–48 h.

#### EEG data processing

The EEG data files were converted into EDF file format and imported into the Brainstorm workstation (https://neuroimage.usc.edu/brainstorm). The 3D individual head model reconstructed by Freesurfer 5.3 was imported simultaneously into the workstation. The MRI registration tool was used to match the EEG leads to the individual head model, and the MRI segmentation tool was used to segment the image and obtain the 3D model of the cortical surface. The EEG of each patient was reviewed by a neurologist with professional EEG training (Z.S. Li and Q.W. Gao), the peaks of the IEDs were marked, and the EEG data between −500 ms and +500 ms at the peaks of the IEDs were averaged. The standardized low-resolution electromagnetic tomography (sLORETA) analysis was performed to locate the source of the average IEDs (14).

#### Interictal epileptiform discharges source connectivity calculation

The phase-locking value (PLV) is an important metric for measuring the synchronization between two time series signals. The algorithm of PLV is also a suitable method for quantifying and analyzing connectivity in dynamic systems and for brain connectivity analysis as a nonlinear dynamic system (15). The synchronicity between two signals in a system can be reflected by the phase angle between the two vectors. PLV is the absolute value of the mean phase difference between the two signals expressed as a complex unit-length vector. The PLV range is 0–1; if the marginal distributions for the two signals are uniform and the signals are independent, then the relative phase will also have a uniform distribution, and the PLV will be 0. Conversely, if the phases of the two signals are strongly coupled, then the PLV will approach 1.

**Figure 1:**
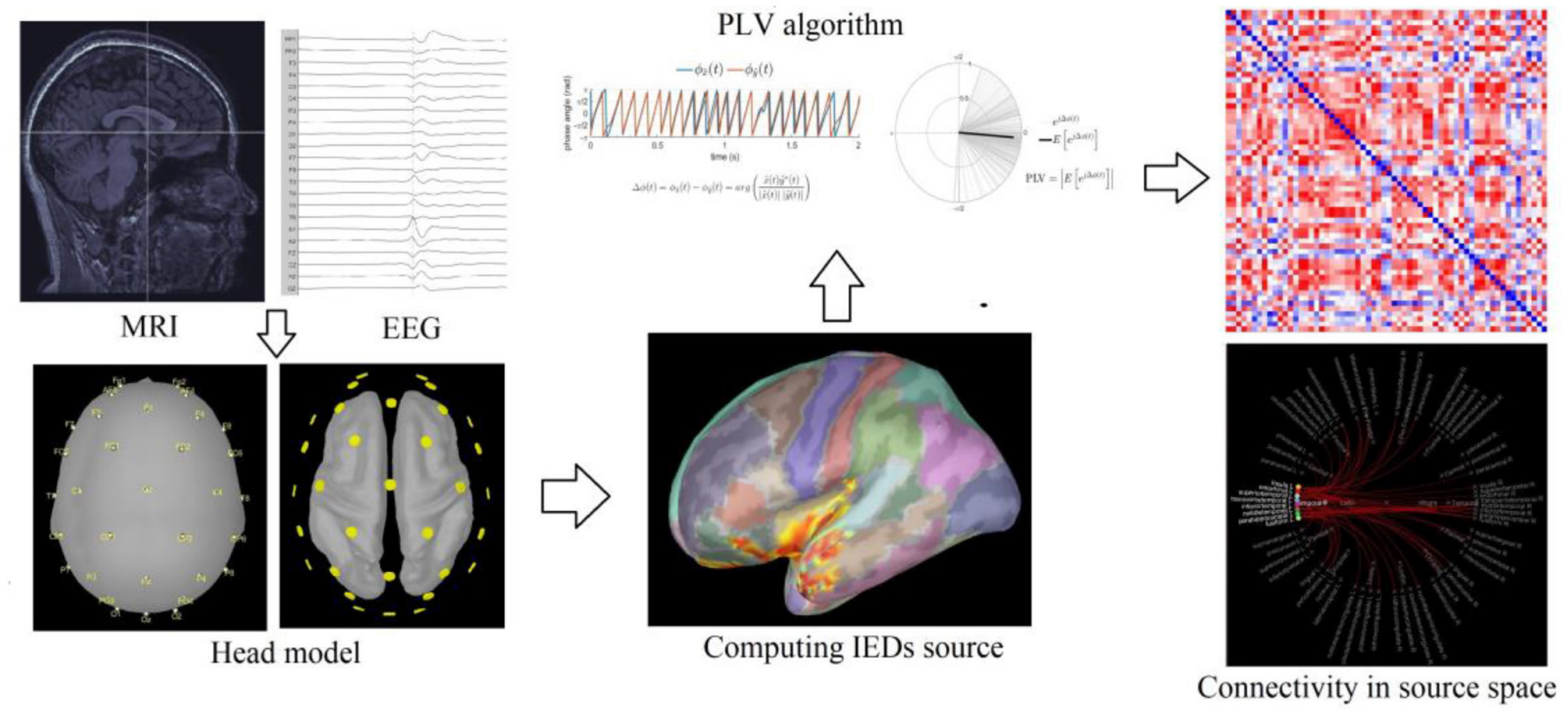
The processing process of IEDs source connectivity.

In our study, the time frame of IEDs ranges from 70 to 200 ms, and the Hilbert transform method of time–frequency transformation is used for the 5.0–14.0 Hz band. The DK parcellations where the source of IEDs was located were used as the nodes, and the connectivity between the nodes was quantified using PLV. The DK parcellations in the temporal lobe (temporal pole, superior, middle, and inferior temporal gyrus, fusiform gyrus, temporal transverse gyrus, entorhinal area, and parahippocampal gyrus) that were ipsilateral to the lesion were selected as regions of interest (ROIs). The PLV values of the IED source between each ROI and other cortical DK parcellations were acquired (Figure 1).

### Statistical analyses

The IBM SPSS 22.0 software package was used for statistical analysis (IBM SPSS Statistics for Windows, Armonk, NY, USA). Descriptive analysis was used for demographic and clinical data characterization. An independent sample *t*-test was used for quantitative data comparison (ages, duration of epilepsy, and ages of onset), and the chi-square test was used for categorical data comparison (sex, seizure type, seizure frequency, number of antiepileptic seizure medicines (ASMs), history of febrile convulsions, and family history of epilepsy). All tests were two-tailed, and significance levels were set at α = 0.05.

Cortical thickness data of each DK parcellation were imported into SPSS v 22.0. Principal component analysis (PCA) was used as a dimension reduction tool to extract the principal components (PCs) that explained 70% of the total variance in the cortical thickness database. Each PC represents a specific cortical structural coupling (SC) due to PCA being a useful technique for exploring connectivity. Finally, we showed the main SCs in a 3D model of the cerebral cortex. We used Spearman's correlation analysis to evaluate the relationship between the PLV of IEDs source in each ROI and the value of each cortical SC.

## Results

### Demographic and clinical characteristics

General demographic information and clinical characteristics, including duration of epilepsy, age of onset, seizure type (focal onset seizures or focal to bilateral tonic–clonic seizures), seizure frequency, number of anti-seizure medications (ASMs), history of febrile convulsions, and family history of epilepsy, were recorded for patients diagnosed with TLE (Table 1).

**Table 1:** Demographic and clinical characteristics of the study participants.

		**Left TLE group**	**Right TLE group**	***t/X^2^***	***P***
Ages		28.03 ± 11.14	28.23 ± 11.86	−0.067	0.947
Sex	Male	22 (66.7%)	10 (38.5%)	4.661	0.031
	Female	11 (33.3%)	16 (61.5%)		
Duration of epilepsy		8.45 ± 6.82	7.96 ± 6.19	0.287	0.775
Ages of onset		19.58 ± 11.79	20.31 ± 12.08	−0.234	0.816
Focal onset seizures frequencies	None	9 (27.3%)	9 (34.6%)	1.722	0.423
	≤ 20 times/year	7 (21.2%)	8 (30.8%)		
	≥21 times/year	17 (51.5%)	9 (34.6%)		
Focal to bilateral tonic clonic seizures frequencies	None	4 (12.1%)	3 (11.3%)	2.675	0.263
	≤ 10 times/year	15 (45.5%)	17 (65.4%)		
	≥10 times/year	14 (42.4%)	6 (23.1%)		
History of febrile convulsions		12 (36.4%)	5 (19.2%)	2.081	0.149
Family history of epilepsy		1 (3.0%)	1 (3.8%)	0.030	0.863
Number of ASMs	None	11 (33.3%)	6 (23.1%)	1.079	0.782
	1 specie	8 (24.2%)	9 (34.6%)		
	2 species	10 (30.3%)	8 (30.8%)		
	≥3 species	4 (12.1%)	3 (11.5%)		

### The important cortical structural couplings in TLE

**Figure 2:**
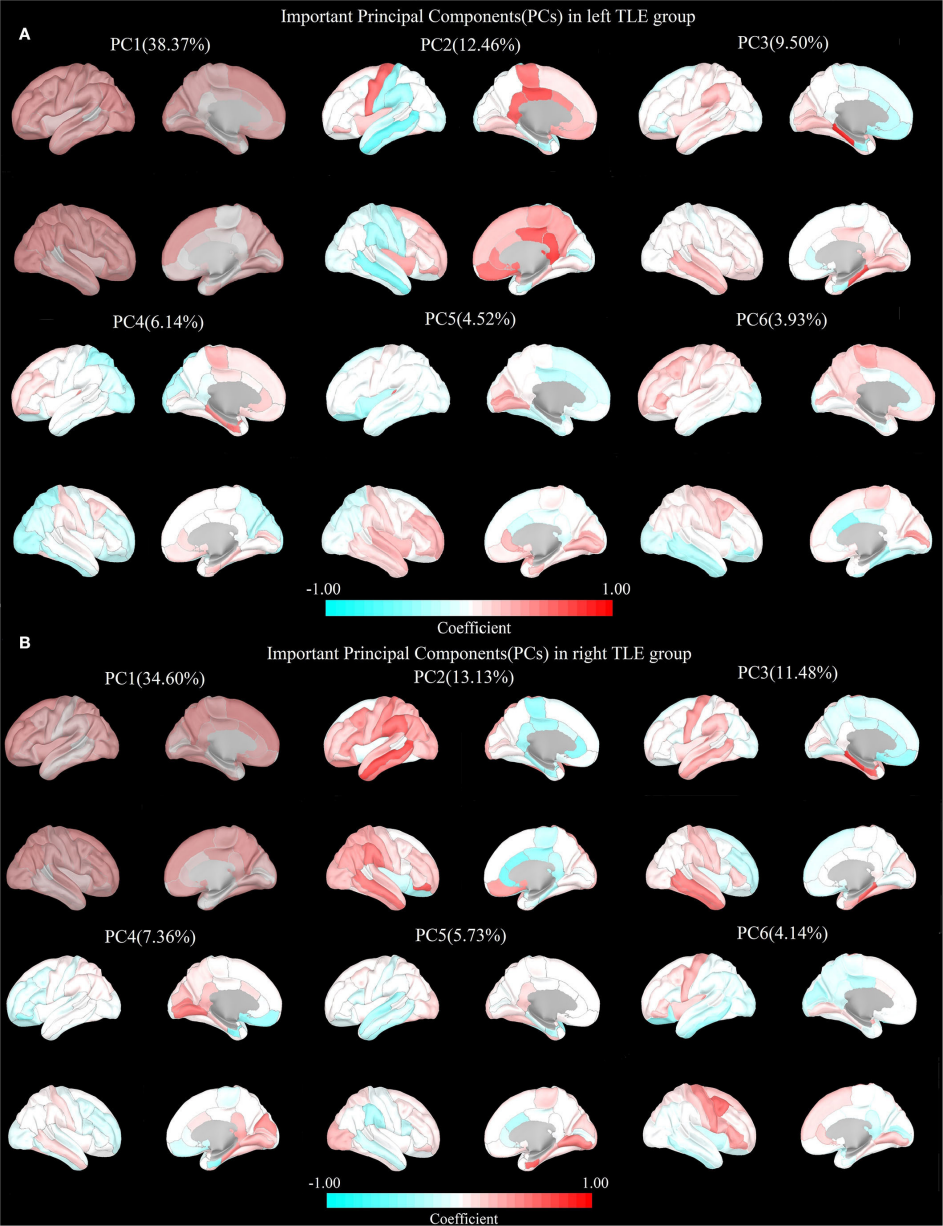
Important principal components (PCs) in both TLEs. **(A)** Left TLE: PC1: Positive distribution in the extensive cortical regions. PC2: Negative distribution in the perisylvian regions. PC3: Negative distribution in the limbic regions. PC4: Negative distribution in the posterior cerebral regions. PC5: Negative distribution in the frontal, insula, cingulate gyrus, and parieto-occipital regions. PC6: Negative distribution in the bilateral temporal lobe. **(B)** Right TLE: PC1: Positive distribution in the extensive cortical regions. PC2: Negative distribution in the limbic regions. PC3: Negative distribution in the frontal, insula, cingulate gyrus, and parieto-occipital regions. PC4: Negative distribution in the bilateral temporal lobe. PC5: Negative distribution in the perisylvian regions. PC6: Negative distribution in the posterior cerebral regions.

A total of six PCs could be extracted from the cortical thickness data of the left and right TLE groups processed by PCA accounting for 74.92 and 76.44% of the total variance, respectively. The distribution of the coefficients in the cortical regions of the four PC pairs in both groups was observably similar, suggesting the consistency of cortical SCs in the two groups. SC1: positive distribution in the extensive cortical regions (PC1 in the left and right TLE groups); SC2: Positive distribution mainly in the posterior limbic regions and negative distribution in the lateral perisylvian regions (PC2 in the left TLE group and PC4 in the right TLE group); SC3: Positive distribution mainly in bilateral medial temporal lobe structures and negative distribution in the anterior limbic regions (both PC3 in the left TLE group and the right TLE group); SC4: Negatively distributed in the ipsilateral insula, nearby cortex, and even ipsilateral hemispheric cortex based on the positive distribution in the extensive cortical regions (PC5 in the left TLE group and PC2 in the right TLE group) (Figure 2).

### The relationship between IED source connectivity and cortical SCs in TLE

**Figure 3:**
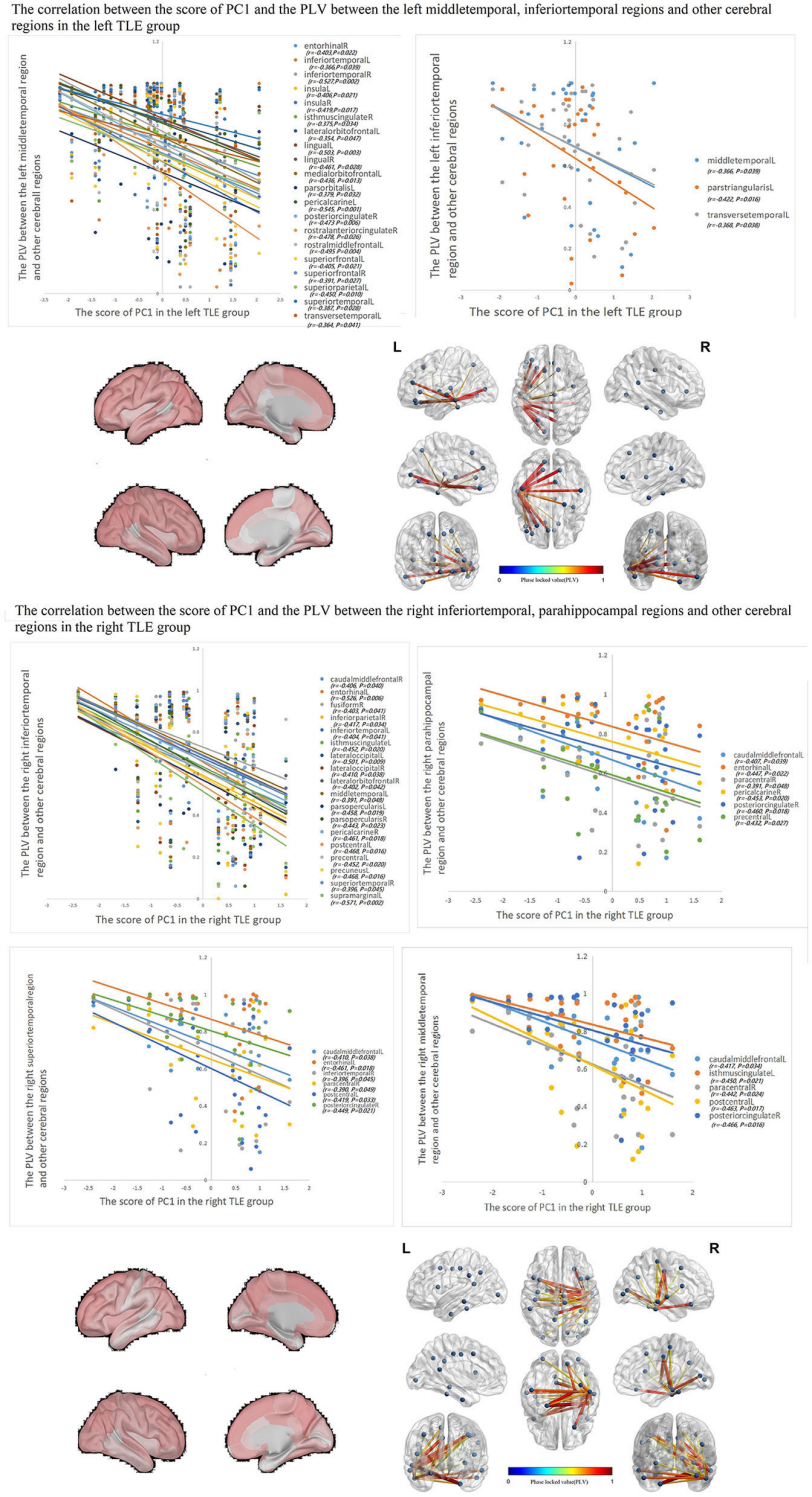
In each TLE group, the IED source connectivity between the ipsilateral temporal lobe and widespread cerebral regions, which included temporal lobes, frontal lobe, insula, cingulate, parietal, and occipital regions, was negatively correlated with SC1.

**Figure 4:**
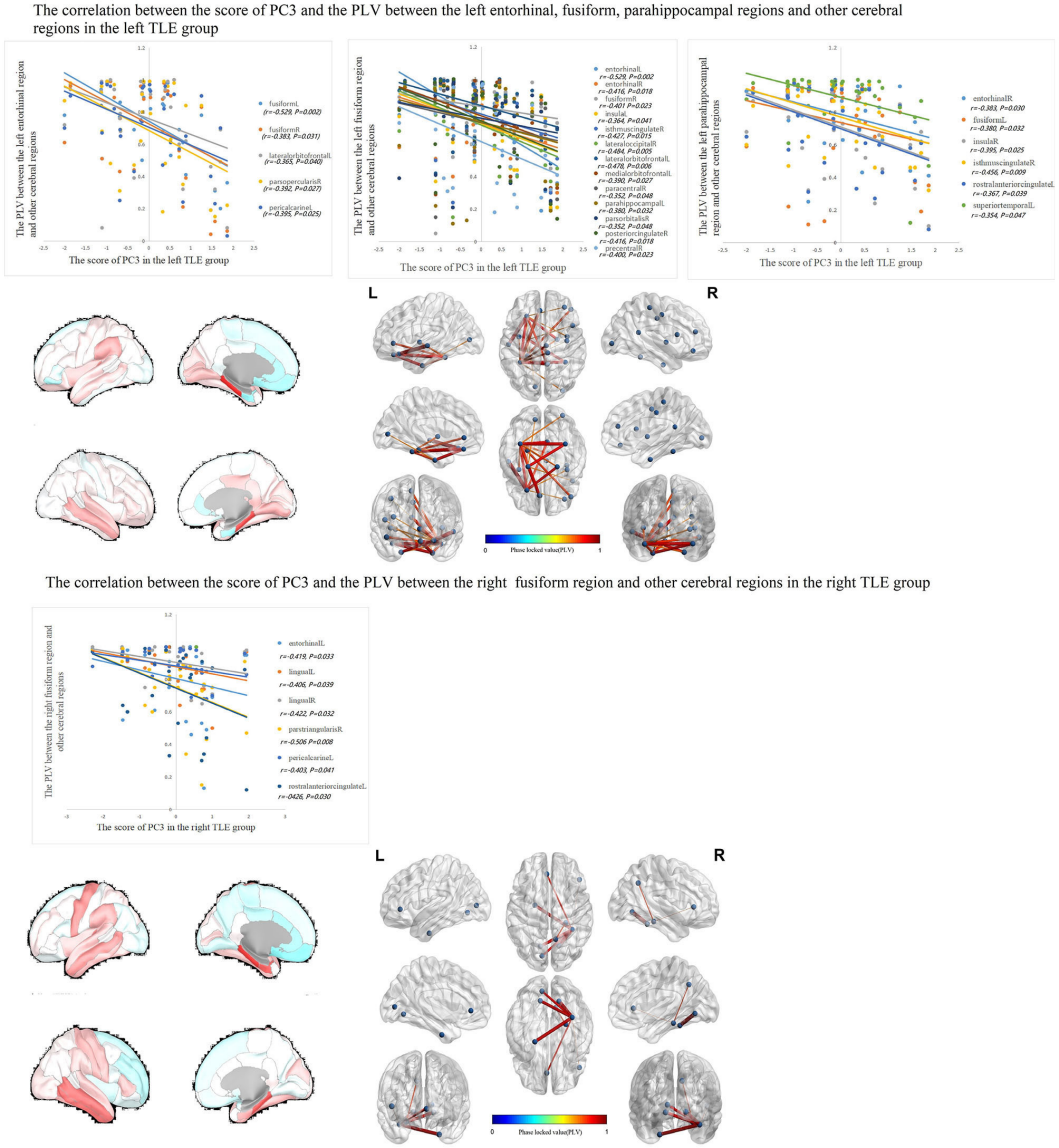
In the left TLE group, the IED source connectivity between the left temporal lobe and the limbic regions which included the medial temporal lobe, cingulate, and orbitofrontal cortex was negatively correlated with SC3. In the right TLE group, the IED source connectivity between the right temporal lobe and the medial temporal and occipital lobes, cingulate gyrus, and ipsilateral pars triangularis cortex was negatively correlated with SC3.

**Figure 5:**
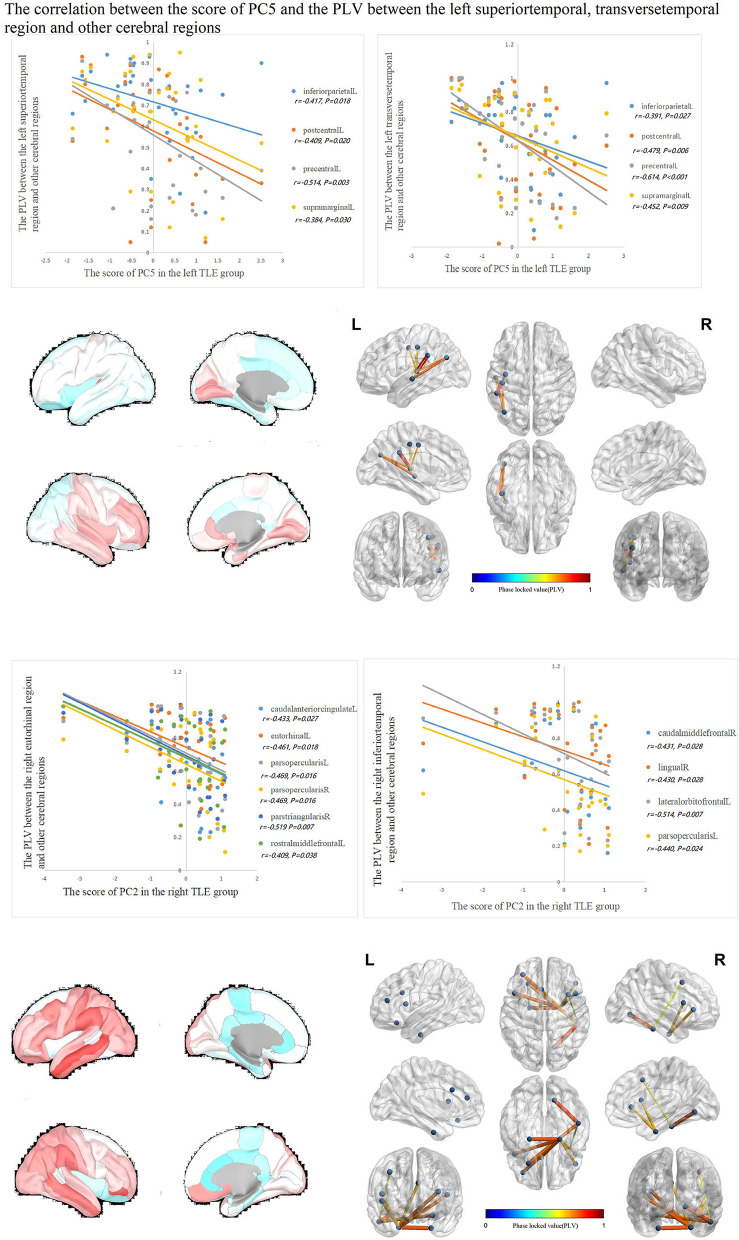
In the left TLE group, the IED source connectivity between the left temporal lobe and the rolandic, parietal cortex was negatively correlated with SC4. In the right TLE group, the IED source connectivity between the right temporal lobe and the frontal, temporal regions was negatively correlated with SC4.

The correlations between IED source connectivity and SCs in both TLE groups showed similarity to some degree, mainly as follows: (1) In each TLE group, the IED source connectivity between the ipsilateral temporal lobe and widespread cerebral regions, which included bilateral frontal, temporal, parietal, and occipital lobes, and insula and cingulate gyrus, was negatively correlated with SC1 (Figure 3); (2) in the left TLE group, the IEDs source connectivity between the left temporal lobe and the limbic regions, which included the medial temporal lobe, cingulate, and orbitofrontal cortex, was negatively correlated with SC3. In the right TLE group, the IED source connectivity between the right temporal lobe and the medial temporal and occipital lobes, cingulate gyrus, and ipsilateral pars triangularis cortex was negatively correlated with SC3 (Figure 4); (3) in the left TLE group, the IED source connectivity between the left temporal lobe and the rolandic, parietal cortex was negatively correlated with SC4. In the right TLE group, the IED source connectivity between the right temporal lobe and the frontal, temporal regions was negatively correlated with SC4 (refer to Figure 5).

## Discussion

In our study, PCA and PLV algorithms were applied to calculate the cortical structural couplings and the IED source connectivity of TLE separately. Correlation analysis was performed to acquire the relationship between the cortical morphology and IEDs in TLE. The results have suggested IED source connectivity, which was negatively related to the important cortical SCs in TLE. To the best of our knowledge, this study is the first attempt to couple the cortical structural and electrophysiological data in patients with TLE to enhance the understanding of the intrinsic cerebral networks using non-invasive methods.

Extensive structural brain atrophy in patients with TLE has been reported in many studies. Illness-associated brain atrophy is a robust finding that remains in effect even after controlling for confounding clinical factors (16). In recent years, studies under the umbrella of the ENIGMA worldwide consortium have mapped the areas of neural atrophy within the prefrontal lobe, the perisylvian regions, the limbic regions, and even subcortical structures such as the thalamus and pallidum in patients with TLE (17). Conceivably, cortical atrophy in these regions can be more pronounced as the disease progresses (18), suggesting that epilepsy might be an independent risk factor for cortical volume decline.

We identified patterns of cortical structural alteration that were reflective of the TLE-related pathology, thus revealing primary substrates in the circuits connecting with the temporal lobe (3, 19). These results agree with the neuroimaging study that employed diffusion tensor imaging (DTI) of the brain; they reported alterations of the white matter fiber tracts, such as the parahippocampal cingulum, fornix/stria terminalis, and the uncinate fasciculus in TLE (20). The categorization of these alterations in TLE in our study also revealed the presence of four important connection groups. The findings from our analyses align with other reports in the literature (21). The importance of cortical SCs lies in the ability to identify important nodes for predicting surgical outcomes and medication response, as well as for uncovering important hub points for later micro-invasive surgery (22, 23). In recent years, it can also be used to reveal the underlying cerebral network mechanisms for the emergence of cognitive function subtypes in TLE. For example, the SC in the perisylvian region is closely related to language function (24). The results suggested the important role of protecting the SCs related to cognitive function in treating TLE. The clinical value of the SC still needs to be further elaborated.

Another highlight of this study is the relationship between IED source connectivity and SCs in TLE. EEG is a commonly used diagnostic method in neurological clinical settings, and due to its high temporal resolution, it is also widely used by researchers to study cerebral functional networks. It is possible to locate the EEG sources and hyperexcitability spreading pathways when applying the inverse algorithms and combining them with imaging data. The electroencephalography source imaging (ESI) was effective in excluding the influence of the confounding impact of volumetric conduction and was even applied to localize the epileptogenic zone (25, 26). PLV was used to evaluate the IED source connectivity here, which could be more confident than the correlation and coherence coefficients. First, we found that the IED source connectivity with the multiple cerebral regions in both TLE groups, especially involving the frontal, insular, and posterior cingulate regions, was negatively correlated with the cortical SC1 of DMN. This result suggests that the wide-spreading of IEDs can cause cortical injuries throughout the brain. The result is similar to the findings of superficial white matter studies that the large-scale decreased functional connectivity in the anterior and posterior DMN hub regions in patients with TLE (27). Other structural and functional connectivity studies based on respective DTI and high-density EEG analysis have also verified that patients with TLE present with decreased structural connectivity efficacy between the temporal lobe and DMN nodes, especially in relation to the decreased connectivity between the temporal lobe and posterior cingulate gyrus (28, 29).

Second, we found that the IED source connectivity between the temporal lobe and limbic regions was negatively correlated with the positively distributed regions in SC3, especially in the bilateral medial temporal lobe and orbitofrontal and posterior cingulate regions. These regions communicated mainly by the anterior commissure and fornix bundle, and epileptic discharges could spread effectively through this pathway in TLE. It was also confirmed by EEG-fMRI studies that IEDs originating from the temporal lobe are often followed by the activation of limbic regions. The cortex in the limbic regions was commonly confirmed by morphological injuries in TLE by numerous studies (30), and the pathological alterations in the limbic circuits were important morphological biomarkers associated with TLE under the use of atypical interhemispheric asymmetry and regional atrophy mapping (31). The degree of disconnectivity in the limbic system was correlated with hippocampal atrophy and abnormal myelin content (32). The disconnection of the medial temporal structures from the limbic system through loss of white matter tracts in the fornix was even confirmed as one of the predicted factors associated with favorable postoperative outcomes (33). These results suggested that the limbic system was an important target for TLE treatment. It would be an important direction for future TLE treatment from the viewpoint of blocking the spreading and injuries of abnormal discharges to the limbic system.

The distribution of SC4 differed from other SCs in that negative distribution in the ipsilateral insula and adjacent cortices were based on DMN. The IED source connectivity that correlated with SC4 was in that diffuse and positively distributed cortical regions, such as the frontal, parietal, central, insula, and occipital lobes cortex, suggesting that the spreading of IEDs through the insula is diffuse and negatively associated with widespread cortical connections. This result was also confirmed in previous studies of IEDs spreading in TLE based on the quantitative analysis of EEG and neuroimaging (10). It could be confirmed that the connection of the insula with extensive brain areas, as well as the connections between the bilateral insula to communicate with the bilateral limbic system in the cortico-cortical evoked potentials study, and the insula has major integrated roles in multimodal functional network processing, such as language, sensation, auditory, visual, limbic, and vestibular functions (34). The involvement of the insula in TLE is commonly viewed as the biomarker of the illness, which can predict poor surgical outcomes (35). The reason was likely to be related to the close connection between the temporal and insular lobe, which could induce widespread dysfunction of DMN by epileptiform activity (36). Hence, this explains our results regarding the negative correlation between the IED source connectivity within bilateral multi-cerebral-lobes with the SC4.

The results of the present study should be viewed in light of several key limitations. (1) The sample size was not large enough, and the variance of all PCs extracted accounts for only 70% of the variance of the overall data, which inevitably leads to a certain degree of bias in the acquisition of SCs. The sample size was needed to increase in future studies and improve the reliability of the results; (2) We only use the cortical thickness data for the cortical analysis in this study, which is not comprehensive enough for the cortical morphological assessment. The other cortical morphological indexes, such as gyrification, and subcortical white matter (SWM) would be involved in our further studies; (3) The correlation analysis was used to explore the relationship between IED source connectivity and SCs. The efficiency of evaluating the causal relationship between the two dimensions was lacking. More appropriate statistical analysis methods were needed.

## Conclusion

This was the first study that performed correlation analysis between the IED source connectivity and cortical SCs in TLE. The negative relationship of IEDs spreading on the corresponding SC in these cerebral regions was confirmed. The results can be used to comprehensively understand the symptomatic manifestations and the comorbidities of TLE from a cerebral structural-electrophysiological connectivity perspective. The MRI-EEG data coupling can potentially be used to identify new therapeutic targets or navigate micro-invasive surgery or neuromodulation therapy.

## Data availability statement

The original contributions presented in the study are included in the article/Supplementary material, further inquiries can be directed to the corresponding authors.

## Ethics statement

The studies involving human participants were reviewed and approved by the Ethics Committee of the General Hospital of Southern Theater Command, PLA. Written informed consent to participate in this study was provided by the participants' legal guardian/next of kin. Written informed consent was obtained from the individual(s), and minor(s)' legal guardian/next of kin, for the publication of any potentially identifiable images or data included in this article.

## Author contributions

ZL and CJ were responsible for the study performance, image processing, statistical analysis, and drafting manuscript. WX and ZQ were responsible for data organization. QG, JL, KP, and WeiW provided patients with TLE and interpreted the data. WeimW and BD contributed to the study concept and design, revising the manuscript, and obtaining funding. All authors contributed to the article and approved the submitted version.
